# Cheminformatics Strategies Unlock Marburg Virus VP35 Inhibitors from Natural Compound Library

**DOI:** 10.3390/v15081739

**Published:** 2023-08-15

**Authors:** Isra M. Alsaady, Leena H. Bajrai, Thamir A. Alandijany, Hattan S. Gattan, Mai M. El-Daly, Sarah A. Altwaim, Rahaf T. Alqawas, Vivek Dhar Dwivedi, Esam I. Azhar

**Affiliations:** 1 Special Infectious Agents Unit BSL3, King Fahd Medical Research Center, King Abdulaziz University, Jeddah 21362, Saudi Arabia; ialsadi@kau.edu.sa (I.M.A.);; 2 Department of Medical Laboratory Sciences, Faculty of Applied Medical Sciences, King Abdulaziz University, Jeddah 21362, Saudi Arabia; 3 Biochemistry Department, Faculty of Sciences, King Abdulaziz University, Jeddah 21362, Saudi Arabia; 4 Department of Clinical Microbiology and Immunology, Faculty of Medicine, King Abdulaziz University, Jeddah 21589, Saudi Arabia; 5 Molecular Diagnostic Laboratory, King Abdulaziz University Hospital, King Abdulaziz University, Jeddah 21362, Saudi Arabia; aalkawas@kau.edu.sa; 6 Bioinformatics Research Division, Quanta Calculus, Greater Noida 201310, India; 7 Center for Global Health Research, Saveetha Institute of Medical and Technical Sciences, Saveetha Medical College and Hospitals, Saveetha University, Tamil Nadu 602105, India

**Keywords:** Marburg virus, VP35, natural compounds, isosilybin, Estradiol benzoate, virtual screening

## Abstract

The Ebola virus and its close relative, the Marburg virus, both belong to the family Filoviridae and are highly hazardous and contagious viruses. With a mortality rate ranging from 23% to 90%, depending on the specific outbreak, the development of effective antiviral interventions is crucial for reducing fatalities and mitigating the impact of Marburg virus outbreaks. In this investigation, a virtual screening approach was employed to evaluate 2042 natural compounds for their potential interactions with the VP35 protein of the Marburg virus. Average and worst binding energies were calculated for all 20 poses, and compounds that exhibited binding energies <−6 kcal/mol in both criteria were selected for further analysis. Based on binding energies, only six compounds (Estradiol benzoate, INVEGA (paliperidone), Isosilybin, Protopanaxadiol, Permethrin, and Bufalin) were selected for subsequent investigations, focusing on interaction analysis. Among these selected compounds, Estradiol benzoate, INVEGA (paliperidone), and Isosilybin showed strong hydrogen bonds, while the others did not. In this study, the compounds Myricetin, Isosilybin, and Estradiol benzoate were subjected to a molecular dynamics (MD) simulation and free binding energy calculation using MM/GBSA analysis. The reference component Myricetin served as a control. Estradiol benzoate exhibited the most stable and consistent root-mean-square deviation (RMSD) values, whereas Isosilybin showed significant fluctuations in RMSD. The compound Estradiol benzoate exhibited the lowest ΔG binding free energy (−22.89 kcal/mol), surpassing the control compound’s binding energy (−9.29 kcal/mol). Overall, this investigation suggested that Estradiol benzoate possesses favorable binding free energies, indicating a potential inhibitory mechanism against the VP35 protein of the Marburg virus. The study proposes that these natural compounds could serve as a therapeutic option for preventing Marburg virus infection. However, experimental validation is required to further corroborate these findings.

## 1. Introduction

The World Health Organization (WHO) has identified Marburg virus (MARV) as a deadly zoonotic pathogen causing Marburg virus disease (MVD) with an 88% case fatality ratio [1]. MARV is a member of the family Filoviridae that is known to cause severe hemorrhagic infections in people [2]. The virus is recognized as having been hosted by the Egyptian rousette bat (ERB), which forms large colonies in caves [3,4,5]. In addition to ERB, MARV has been found in the green monkey *Chlorocebus* sp. and insectivorous bats that inhabit caves, such as *Miniopterus inflatus* and *Rhinolophus eloquens* [6,7,8]. Moreover, the Angola-like variant of the virus gained recognition following the largest outbreak of MARV, which occurred in Angola (2005) and resulted in 252 cases with 227 fatalities [9]. In addition, equatorial Guinea has observed an upsurge in Marburg virus disease (MVD) cases, with an additional six laboratory-confirmed cases documented on 22 March 2023 (based on data collected by 21 March 2023) [10]. Since the beginning of the outbreak on 13 February 2023, there have been a total of 15 instances of MVD that have been confirmed by laboratories, in addition to 23 cases classified as probable cases [11]. The province of Litoral’s Bata is the area that has been the most affected by the virus, with nine instances of MVD that have been confirmed by a laboratory. Eleven people who had their illnesses confirmed by a laboratory have since passed away, while the prognosis for the remaining confirmed cases is unknown [11].

There is currently no vaccination or medicine that has been shown to be effective in preventing the disease caused by the Marburg virus. This is the case even though research into the subject has been ongoing for over 50 years. On the other hand, despite their history of therapy for Ebola sickness, the antiviral medications favipiravir and remdesivir could potentially be utilized to treat Marburg virus disease in the absence of any other well-established therapeutic alternatives [12]. Therapeutic compounds for antiviral medications, monoclonal antibodies, vaccines, cytokines, host-targeted therapeutics, and drugs that are attempting to treat pan-filovirus infections are all included in treating MARV [13,14]. The Marburg virus can be passed on from one human to another through exposed skin or mucous membranes, as well as through direct contact with the blood, organs, secretions, or other bodily fluids of an infected person. The virus can also be passed on through surfaces and materials (such as bedding, clothing, and other textiles) that are contaminated with these fluids [1]. Moreover, filoviral infections are pantropical, which means they can affect almost every organ of the infected individual.

The Marburg virus envelope consists of seven structural proteins and non-segmented negative stranded RNA [15]. The nucleocapsid is composed of four essential proteins: the L protein, VP30, VP35, and nucleoprotein. Each of these proteins is located on a different subunit of the nucleocapsid (NP). VP35, NP, and L are crucial components of the virus that must be present for viral transcription and replication to take place. The viral protein known as VP35 is necessary for the assembly of the nucleocapsid, the replication of the virus, and the transcription of the virus. Additionally, VP35 plays an important role in the immunosuppression of the host [16]. VP35’s capacity to bind to viral dsRNA induces antagonism of the type I IFN response, inhibits phosphorylation/activation of interferon regulatory factor 3 (IRF-3), and suppresses RNA silencing. This binding also masks VP35 from host factors and likely prevents its degradation [17,18,19,20,21]. Additionally, various studies have shown that VP35 can be used as a potential drug target for MARV as it is effective in Ebola infection treatment [22,23].

In this study, computational approaches were employed to identify promising compounds with potential anti-Marburg activity. In this study, natural compounds were virtually screened for their effectiveness against the target protein, which was VP35 from the Marburg virus. The resulting docked complexes were thoroughly analyzed, considering binding energies and interactions, to identify the most promising compounds. Following this, selected compounds were run through molecular dynamics simulations, and myricetin was used as the control compound in a comparative study. In addition, the MM/GBSA technique was utilized in order to ascertain the free binding energies of the compounds that were selected. Collectively, these findings highlight potential candidates that could effectively target VP35 of the Marburg virus, offering promising avenues for the development of therapeutic interventions against this disease.

## 2. Materials and Methods

### 2.1. Protein Structure and Screening Library

The viral protein known as VP35 from the Marburg virus was selected as the therapeutic target protein for this in-silico investigation. The crystallized structure of VP35 was obtained from the online database, protein data bank (PDB) with PDB ID: 4GH9 [24]. In this case, the natural substances were collected from the Selleckchem database, which may be accessed at https://www.selleckchem.com/, accessed on 15 May 2023. After collecting a total of 2569 compounds, filters were applied to include only those with a molecular weight (MW) of 500 Daltons or less. These selected compounds underwent virtual screening in relation to the target protein, VP35.

### 2.2. Virtual Screening

The drug design process is divided into virtual screening, pose prediction, and binding energy calculation. Drug-like compounds for a given target can be identified through a process known as virtual screening, or in-silico screening [25]. In order to perform massive simulations and calculations, virtual screening takes advantage of recent developments in computational power and algorithms. Molecular docking simulations, pharmacophore-based screening, similarity searches, and more complex procedures involving artificial intelligence are all examples of the types of computational methods used in drug discovery [26]. Docking is recognized as a highly promising approach in the field of structure-based drug design [27], thus used in this investigation. In this study, performing virtual screening aimed at finding compounds that could interact with and modulate the activity of the target protein, potentially leading to therapeutic effects. The obtained natural compounds were docked by employing AutoDock Vina [28].

The AutoDock Vina molecular docking program is one of the most popular and widely used docking methods currently available. A fundamental scoring method and a rapid gradient-optimization conformational search from the foundation of a comprehensive computational docking program. The scoring function is based on the conformation of the protein, calculating the orientation that a compound is most likely to take when it is attached to a protein [29]. The CASTp web server was utilized to identify the protein’s binding pocket [30]. The CASTp web server, which is a tool for identifying geometric and topological features, can be used to make predictions regarding the binding cavity of the protein structure. These predictions can then be tested and validated. The CASTp server was queried with chain A of PDB ID: 4GH9, and the highest-ranking binding pocket was selected as the target. The residues revealed to be located in the binding pockets were used to construct the grid for the virtual screening procedure. The target protein crystal structure, VP35 of the Marburg virus, was docked with the selected natural compounds. The grid box was created for docking the compound with dimensions of 35 Å each in X, Y and Z directions. The residues that covered the binding sites were centered at 15.841 Å, 17.832 Å, and 9.839 Å. The AutoDock Vina tool was used to perform the molecular docking screening on these molecules. Different parameters were fixed to certain values before executing the docking. The binding mode parameter that counts the number of poses generated at the end of the docking was set at 20 during the run, while exhaustiveness, which indicates the amount of geometrical space covered, was set at 10. The maximum energy difference was at 4 kcal/mol for the different poses. Each of the compounds that had been selected was eventually docked within the binding site of VP35, and the best hits were then put through additional investigation.

In addition, the compounds went through a filtering process based on the average binding energy they possessed. After that, they were chosen based on the lowest binding energy. In this part of the process, the average binding energy of each of the compounds was determined, and then they were ordered accordingly. The potential binding dock energy for active drugs is traditionally considered to be −6 kcal/mol [31,32,33]. Thus, this binding score for standard drugs greater than or equal to −6 kcal/mol was set as the threshold. This threshold signifies a predicted strong interaction between a potential drug candidate and its target protein. Using a threshold like this helps to filter out compounds that are likely to bind weakly or nonspecifically, which can lead to false positives. Previous studies have also indicated that binding energies of <−6 kcal/mol are taken as a standardized rule of selection; thus, this was applied in this study to identify potential binders [34]. Based on the average binding energy ranking technique, the compounds having an average binding energy (across all the poses generated in the docking) of <−6 kcal/mol were selected. Later, the binding energies of the last pose (worst binding energy) of the selected compounds were considered. Moreover, compounds whose binding energy is <−6 kcal/mol, even in their worst pose, are considered to have a maximum chance of having a high binding affinity for the target protein. Thus, the compounds having the worst binding energy of <−6 kcal/mol were selected further.

Subsequently, acquired compounds were subjected to interaction analysis using BIOVIA Discovery Studio. Discovery Studio is a suite for the simulation of small molecule and the macromolecule of the systems [35]. BIOVIA Discovery Studio offers a range of tools for conducting interaction analysis between ligands, such as potential binders and protein targets. This analysis aims to understand the binding interactions, orientations, and energies of ligands within the protein’s active site. Ligand-protein interactions were analyzed using BIOVIA Discovery Studio’s interaction analysis tools. Hydrogen bonds, hydrophobic contacts, and other non-covalent interactions were identified within each ligand pose. Hydrogen bonds were characterized by considering the distance and angle criteria, while hydrophobic contacts were defined based on van der Waals interactions. Molecular visualizations were generated to depict the specific amino acid residues and ligand atoms involved in interactions, aiding in the interpretation of binding modes. Upon analysis of the interactions, only the compounds exhibiting hydrogen bond interactions were selected. Hydrogen bonding plays a critical role in the docking values as it significantly contributes to the structural aspects of drug development [36]. Furthermore, the obtained compounds were subjected to molecular dynamics simulation and MM/GBSA to study the stability and flexibility of protein-ligand complexes.

### 2.3. Molecular Dynamic Simulation

The molecular dynamic (MD) simulation was conducted with the chemicals that were selected. MD simulation is a computational approach that examines the behavior and interactions of atoms and molecules over time. It involves solving the equations of motion for a system of particles, typically using classical mechanics. The aim is to replicate the actions of the system over a specific duration [37]. Here, GROMACS-2021 applications were employed to perform molecular dynamics simulations on selected complexes, employing the CHARMM36 force field for force dynamics. The CGenFF software was employed for the purpose of generating topologies and compatible parameters that are in accordance with the CHARMM all-atom force field. This facilitates the preparation of small molecules by adding appropriate parameters to the atoms of the compound [38]. Electrostatic calculations were performed using the Ewald Particle Mesh method [39]. Following the solvation of the simulation box applying the TIP3P water model and subsequent neutralization through the introduction of Na^+^ and Cl^−^ ions, the simulation was executed. The protein and the ligand made up the solvated complex, which was placed in the middle of the solvated box while maintaining 1 Å from the wall of the solvated box. This was followed by a total of 5000 steps with the steepest descent algorithm for minimizing the complex energetically. The minimized system was heated to a temperature of 310 K [40]. The system has been brought to equilibrium for two different ensembles of constant temperature (NVT) and constant pressure (NPT) by maintaining a temperature of 310 K and a pressure of 1 bar for a timescale of 1 ns each. The equilibrated system was used in the 100 ns production cycle. The velocity-rescaling technique was used for temperature coupling [41], while using the Parrinello-Rahman pressure method to maintain pressure [42]. Additional parameters and settings for MD were listed in the Supplementary File Table S1. The conformational analysis was conducted employing the GROMACS-provided essential metrics.

### 2.4. Binding Free Energy (MM/GBSA)

The Molecular Mechanics Generalized Born Surface Area (MM/GBSA) technique was applied to perform the necessary calculations for determining the binding free energy of the protein-ligand complex. This was accomplished by running the gmx MMPBSA program [43,44]. The last 20 ns of the molecular dynamics simulation trajectory were used for estimating the GTOTAL binding free energy for the chosen compounds. The solvation parameter, also known as igb, was assigned a value of five, while the concentration of salt in the system was set at 0.154 M. The default values of 1.0 and 80.0 were assigned to additional parameters, namely the internal and external dielectric constants. Equation (1) illustrates the application of the MM/GBSA calculation methodology:

(1) $$\Delta G=G_{complex\;}-[\;G_{receptor}+G_{ligand}]$$

The value of the complex’s, the receptor’s, and the ligand’s free energy that was acquired from the simulation trajectory frames during the most recent 20 ns is represented by Equation (1). Equations (2)–(6) are used to determine the energy components that are incorporated into the calculation of ΔG:

(2) $${\Delta G}_{binding}=\;\Delta H\;-\;T\Delta S$$

(3) $$\Delta H\;={\;\Delta G}_{\mathrm{GAS}}+{\;\Delta G}_{\mathrm{SOLV}}$$

(4) $${\Delta G}_{\mathrm{GAS}}={\;\Delta E}_{\mathrm{EL}}+{\;\Delta E}_{\mathrm{VDWAALS}}$$

(5) $${\Delta G}_{\mathrm{SOLV}}={\;\Delta E}_{\mathrm{GB}}+{\;\Delta E}_{\mathrm{SURF}}$$

(6) $${\Delta E}_{\mathrm{SURF}}=\;\gamma .\mathrm{SASA}$$

In addition, the enthalpy change ΔH includes the summation of solvation free energy (ΔG_SOLV_) and gas-phase energy (ΔG_GAS_). In this context, TΔS denotes the entropy contribution made by the free binding energy. Here, electrostatic and van der Waals terms combine to generate ΔG_GAS_ (represented by ΔE_EL_ and ΔE_VDWAALS_, respectively). The total solvation energy ΔG_SOLV_ is composed of both the polar solvation energy, denoted by ΔE_GB_, and the nonpolar solvation energy ΔE_SURF_. In this case, ΔE_SURF_ was computed by taking the product of the area of solvent-accessible surface (SASA) and the parameter representing solvent surface tension (γ). Subsequently, the simulation trajectory was transformed into a format compatible with Bio3D [45,46] for conducting principal component analysis (PCA) [47]. The R programming language was utilized for this purpose. In this analysis, the initial coordinates of the molecule served as a point of reference, and the subsequent conformations produced during the simulation were aligned with them to calculate the eigen vector. This vector represents the orthogonal principal components of motion exhibited by the molecule.

Additionally, the hit compound selected as a potential VP35 inhibitor was analysed for physicochemical properties and pharmacokinetics parameters using the SwissADME [48] and ProTox-II server [49].

## 3. Results and Discussions

### 3.1. Binding Pocket

**Figure 1:**
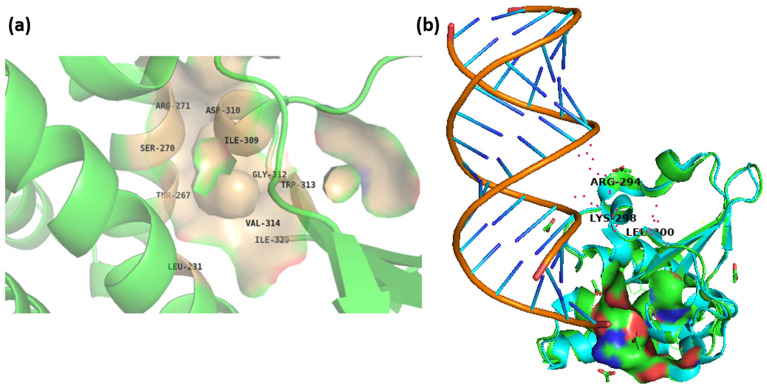
Top rank (**a**) binding pocket of 4GH9 (VP35) predicted by CASTp web server with the key residues (**b**) binding site residues shown as surface along with conserved residues Arg294, Lys298, and Leu300 that bind with the dsRNA molecule.

The CASTp online server was employed to predict the binding pocket of the viral protein VP35 of the Marburg virus with the PDB ID: 4GH9, and the residues were specified so that the binding grid box could be determined for the purpose of the virtual screening procedure. The largest binding pocket, comprising Leu^231^, Thr^267^, Ser^270^, Arg^271^, Ile^309^, Asp^310^, Gly^312^, Trp^313^, Val^314^, and Ile^329^ of VP35 was chosen for virtual screening. The residues on VP35 expected to be binding sites were used in the construction of the grid box for the virtual screening technique. Figure 1a displays the binding pocket that was predicted by the CASTp server for the VP35 protein. The CASTp server’s estimation of the protein’s binding site showed that it contained a sizeable region that encompassed the protein’s most important binding pocket, as is illustrated in Figure 1a. The grid box, built with these binding site residues, was used for site-specific docking. The predicted binding site, identified as having a cleft shape by CASTp, is crucial for the binding of small compounds. Interestingly, certain residues (Arg294, Lys298, Leu300) found in the Ebola virus have been implicated in binding to dsRNA (PDB Id: 3KS8), and these residues are conserved in the Marburg virus as well. Although these residues do not directly form part of the binding site, they contribute to a flat surface that can interact with RNA molecules, albeit not deeply enough to accommodate small compounds. Nonetheless, due to their proximity to the predicted binding site, these residues are likely to undergo alterations upon binding with a ligand, as shown in Figure 1b.

### 3.2. Virtual Screening

**Figure 2:**
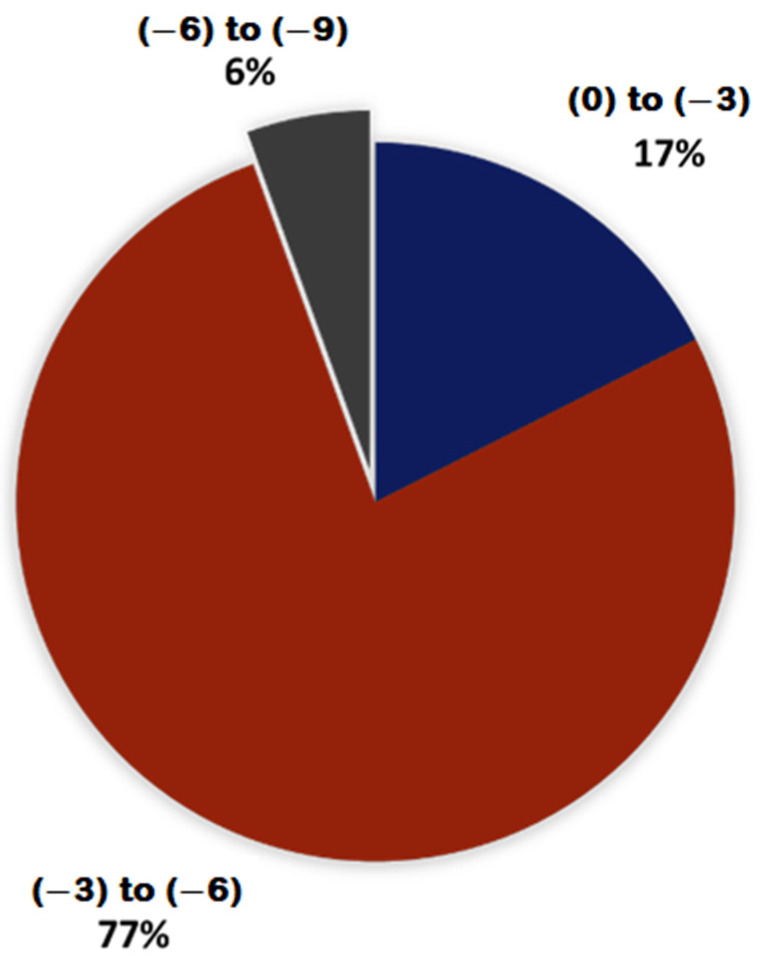
Pie chart for the binding energies of the 2042 natural compounds docked against the VP35 of Marburg virus. Here, the 6% depicted in grey had binding energies of <−6 kcal/mol.

**Figure 3:**
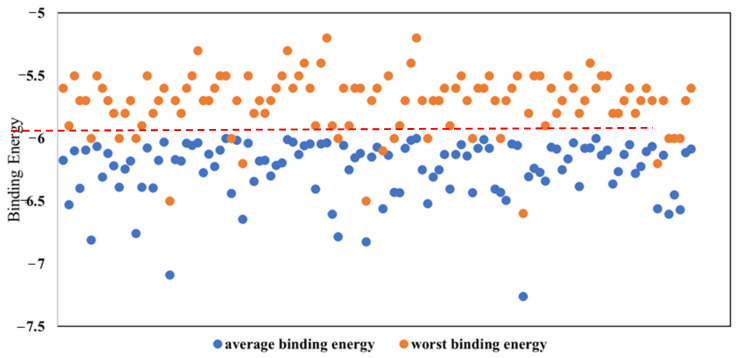
Scattered plot for the average binding energies and the worst binding energies of the 2042 natural compounds docked against the VP35 of Marburg virus.

In this study, a total of 2569 natural compounds were collected from the Selleckchem database for the purpose of conducting virtual high-throughput screening. It was observed that some compounds exceeded the size of a standard drug molecule and may not bind to small target proteins, such as VP35. Therefore, a criterion was used to select only the small compounds for screening. Specifically, only compounds with a molecular weight of 500 Dalton or less were selected, which resulted in 2042 natural compounds eligible for virtual screening against the VP35 protein of the Marburg virus. In addition, the molecular weight criterion also enhances their potential as suitable drug candidates in later stages. Thus, a total of 2042 compounds were chosen for molecular docking using the Autodock Vina program, with the VP35 RNA binding domain of the Marburg virus selected as the receptor. A docking exhaustiveness of 10 was employed during the molecular docking process. The average binding energies were calculated, and the compounds were accordingly ranked. In a previous investigation, possible binders were determined to be substances that had binding energies that were lower than −6 kcal/mol [34]. Therefore, compounds with binding energies below −6 kcal/mol were selected, resulting in a total of 113 compounds screened for further investigation, as shown in Figure 2. Out of the total compounds that underwent docking, only 6% were sorted based on their worst binding energies (i.e., the binding energy of the last pose). Among these, compounds that exhibited the worst binding energy below −6 kcal/mol (6 compounds) were chosen for the subsequent phase of investigation. Both the average and worst binding energy criteria were used to select compounds capable of binding effectively even in the poorest position. Figure 3 presents the average and worst binding energies of the compounds. Eventually, the docked complexes of the six selected compounds (Estradiol benzoate, INVEGA (paliperidone), isosilybin, protonaxdiol, permethrin, and bufalin) were further selected and investigated for their interactions.

### 3.3. Interaction Analysis

**Figure 4:**
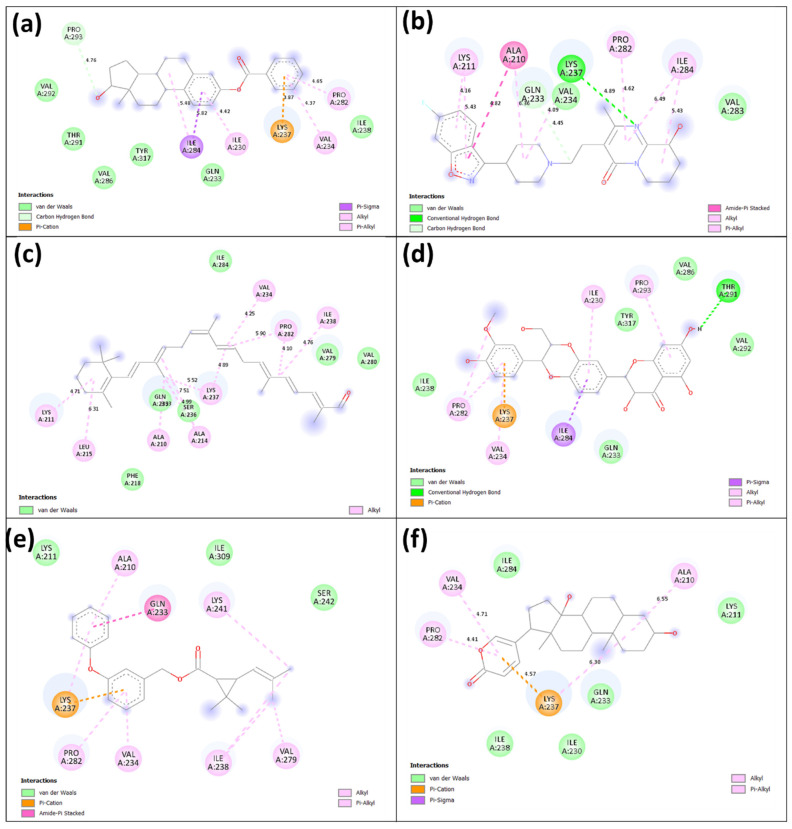
Intermolecular interaction represented in 2D for the compounds (**a**) Estradiol benzoate, (**b**) INVEGA (Paliperidone), (**c**) Protopanaxadiol, (**d**) Isosilybin, (**e**) Permethrin, and (**f**) Bufalin with the target protein VP35 of Marburg virus.

Interaction analysis is essential in molecular modelling and drug discovery to understand the complex interactions between biomolecules like proteins and ligands. This study examines the spatial arrangement, energetic contributions, and structural characteristics of interactions that form and stabilize molecular complexes. The intermolecular interactions within the docked complexes were shown using BIOVIA Discovery Studio for the best pose of the six selected compounds. Figure 4 visually represents the 2D interaction plot observed in these complexes. The analysis showed the presence of multiple types of bonds, including van der Waals, π-cation, π-alkyl, amide-π stacked, π-sigma, and alkyl interactions. On the other hand, it is essential to keep in mind that hydrogen bonds play a significant role in the protein-ligand interactions that take place within the docked complexes. These interactions have substantial implications for structure-based drug development [36]. These hydrogen bonds are essential for ensuring stability and specificity in protein-ligand complexes and can greatly influence the binding affinity and selectivity of the ligand for its target protein. Among the selected compounds, Estradiol benzoate, INVEGA (Paliperidone), and Isosilybin were found to form hydrogen bond interactions, while Protopanaxadiol, Permethrin, and Bufalin did not form any hydrogen bonds. Specifically, as shown in Figure 4a,b,d, Estradiol benzoate formed a single carbon-hydrogen bond (covalent interaction) with the Pro^293^ residue, whereas INVEGA (Paliperidone) and isosilybin formed a single conventional hydrogen bond (non-covalent interaction) with the Lys^237^ and Thr^291^ residues of the VP35 protein, respectively. Binding of hit compounds at the critical site is the preliminary stage in the inhibition process; by competing with the natural ligand, it can reduce the activity of a protein. Here, Estradiol benzoate, INVEGA (Paliperidone), and isosilybin demonstrated higher binding at the active site of the protein and were therefore selected for further MD simulation.

### 3.4. Molecular Dynamics (MD) Simulation

Molecular dynamics is a technique for determining the motion of biological and chemical systems using physics-based principles. Earlier investigations in this study utilized a rigid-body approach, whereas MD provides flexibility and simulates their dynamics. This resulted in the widespread use of MD simulation for in-silico drug design [50]. It can estimate the stability of protein-ligand complexes and determine their binding affinities. In this study, 100 ns timescale MD simulations were carried out for each complex, and their trajectories were analyzed to calculate the root mean square deviation (RMSD) and root mean square fluctuation (RMSF) to ascertain their conformational behavior. Subsequently, hydrogen bonds and binding free energy calculations were conducted to determine the ligand’s affinity for the target protein.

#### 3.4.1. RMSD

**Figure 5:**
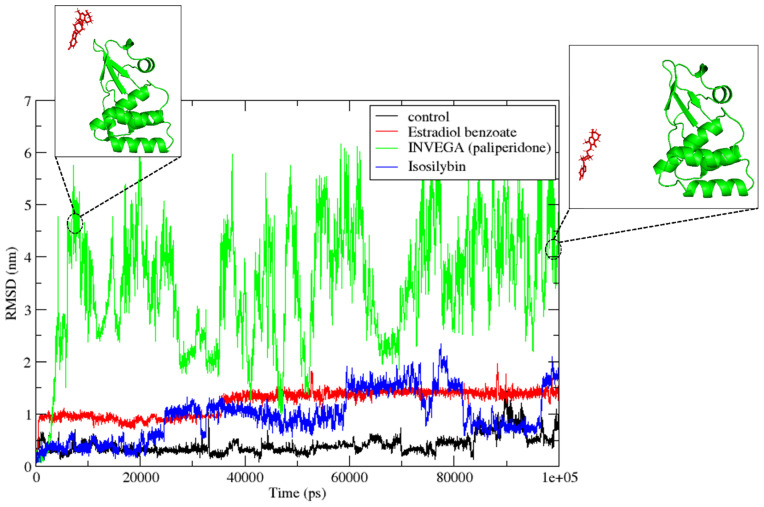
Ligand RMSD plot for the compounds Estradiol benzoate, INVEGA (Paliperidone), Isosilybin along with the control myricetin. The protein-ligand conformation at 5 ns and 100 ns of the simulation for the compound INVEGA (Paliperidone) has also been illustrated.

In a standard MD simulation, the root mean square deviation is derived from the equilibrated structure, which confirms the conformational variation of the protein and ligand molecules. Protein conformation may vary upon ligand interaction, reflecting the effect of binding. Calculating RMSD is dependent on the molecule being aligned; the RMSD of the ligand was computed after aligning the protein residues. This measures the rotational and translational motion of the ligand within the protein’s binding pocket. After rigid docking, the docked pose of the ligand was obtained. Therefore, there is a significant possibility that the ligand will find a better site during MD simulation. The least energy state can be attained via rotational and translational motion. To compare the deviation of the hit molecule with the deviation of the standard molecule, the RMSD of the control compound was also determined. As indicated in Figure 5, RMSD was determined for each frame after 100 ns of simulation. In this situation, INVEGA (paliperidone) showed a higher RMSD than other hit compounds. This compound exhibited a significant displacement of 5 nm from its initial docked position, indicating its dissociation from the protein molecule.

Moreover, it was observed that the compound INVEGA (paliperidone) moved outside the protein binding region throughout the 100 ns simulation, indicating weak binding of INVEGA (paliperidone) towards the target protein VP35. Therefore, INVEGA (paliperidone) was excluded from the list of selected compounds for further investigation. The compounds Isosilybin and Estradiol benzoate, along with the control myricetin, were used for protein and ligand RMSD calculations, respectively.

**Figure 6:**
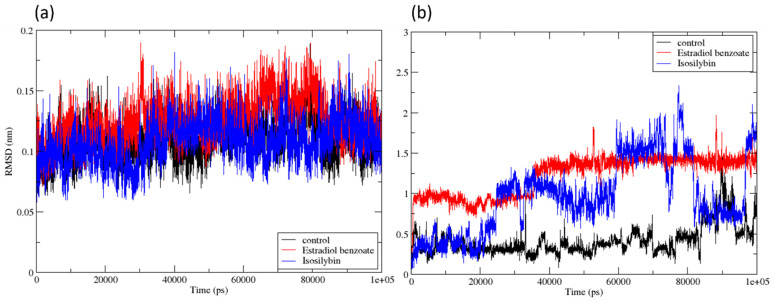
RMSD for the compounds Estradiol benzoate and Isosilybin along with control, Myricetin, (**a**) RMSD of protein Cα-atoms aligned over the initial structure (**b**) RMSD of ligand aligned over the initial structure of protein.

As observed in Figure 6a, the root mean square deviation of the protein was determined for the 100 ns simulation. Notably, the RMSD of the protein bound to the compounds was found to be stable and consistent, ranging from 0.1 nm to 0.2 nm, with no major fluctuations in this value. Throughout the course of the 100 ns simulation, the root mean square deviation (RMSD) of the ligands was determined (Figure 6b). Myricetin, the control compound, displayed the least variance in its RMSD for the first 80 ns, with RMSD values that were continually lower than 0.5 nm. Nevertheless, in the final 20 ns of the trajectories, the RMSD increased from 1 nm to 1.5 nm, which showed a conformational shift in the ligand. In contrast, the molecule Estradiol benzoate displayed a remarkably stable and consistent RMSD with only minor changes. The RMSD stayed stable at around 1 nm during the first 40 ns of the simulation. Thereafter, the RMSD steadily climbed to 1.5 nm for the duration of the simulation without experiencing any notable variations. Although this suggested that the ligand’s binding site had shifted, the strength of the connection between the ligand and the protein remained unaffected. Isosilybin, on the other hand, exhibited more dramatic changes in RMSD during the course of the 100 ns simulation. After an initial period of 0.5 nm, which lasted for the first 20 ns, the RMSD of the ligand increased to 1 nm, where it remained steady until 60 ns. Following that, the RMSD progressively increased to 1.5 nm to 2 nm, fluctuated for the next 80 ns, then suddenly reduced to 0.7 nm before abruptly increasing to 1.5 nm once again. These RMSD fluctuations of Isosilybin indicated that the binding between the compound and the protein was inconsistent during the 100 ns simulation. Conversely, Estradiol benzoate exhibited a consistently stable binding with the protein for the majority of the simulation duration. The initial conformation of the ligand shifted by 1.5 nm compared to its position in the docked state. This suggested that the docked conformation of the ligand was not very stable and it searched for new conformations within the binding site. However, once it reached a state that was substantially more stable, it remained in that state until the end of the simulation.

#### 3.4.2. Conformation of Protein-Ligand Complex

**Figure 7:**
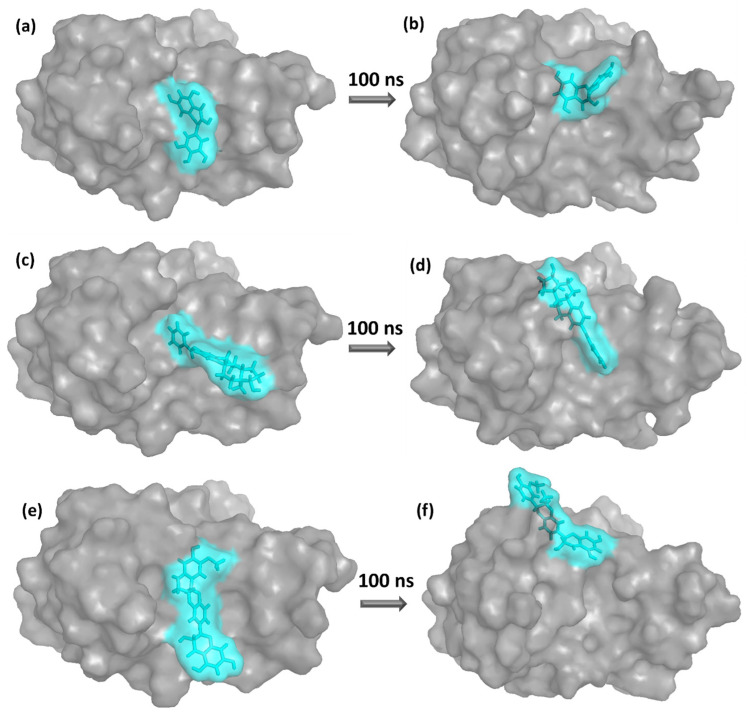
Initial and final poses of the protein-ligand complexes after the 100 ns MD simulation for (**a**,**b**) Myricetin (control) (**c**,**d**) Estradiol benzoate (**e**,**f**) Isosilybin.

The 3D structures of the first and last poses of the selected ligands, along with the control (Myricetin), were illustrated in Figure 7. Notably, it was evident that the final poses of all the ligands exhibited significant deviations from their initial poses. Specifically, the initial binding site of the control ligand (Myricetin) within the protein’s binding pocket was preserved even in the final pose, as illustrated in Figure 7a,b. However, both translational and rotational motions were observed in the final pose of the control compound. Likewise, the chemical structure Estradiol benzoate exhibited identical binding behavior when the simulation first started, and it continued to be bound within the same binding pocket of the protein for the entirety of the MD simulation. However, there were noticeable conformational changes compared to its initial state. Insights into the interaction between the protein and the ligand, as well as the mobility of the ligand compound within the binding site of the protein were immediately shown by comparing the initial pose to the final pose that occurred during the simulation. These inferences were made possible by observing the difference between the two poses. Estradiol benzoate did not exhibit significant translational displacement in its final pose, indicating binding stability (Figure 7c,d). In contrast, the compound Isosilybin showed relatively greater translational motion compared to its initial pose in Figure 7e,f. Rotational movement was observed in all cases, where the ligand compounds adjusted their rotational bonds and positioned their functional groups to achieve optimal interactions.

Overall, Figure 7 demonstrated the stability of these compounds within the protein’s binding cavity. However, it is important to note that the 3D representation of the protein-ligand complex in the initial and final poses cannot accurately quantify the magnitude of movement. The initial and final poses shown in Figure 7 have demonstrated the change in ligand conformation, indicating the search limitation of the docking program. However, MD simulation can capture the stable position that the ligand achieved in the simulation.

#### 3.4.3. RMSF

During a molecular dynamics (MD) simulation, the Root Mean Square Fluctuation (RMSF) is a measure that is used to examine the flexibility or mobility of individual residues in a protein.

**Figure 8:**
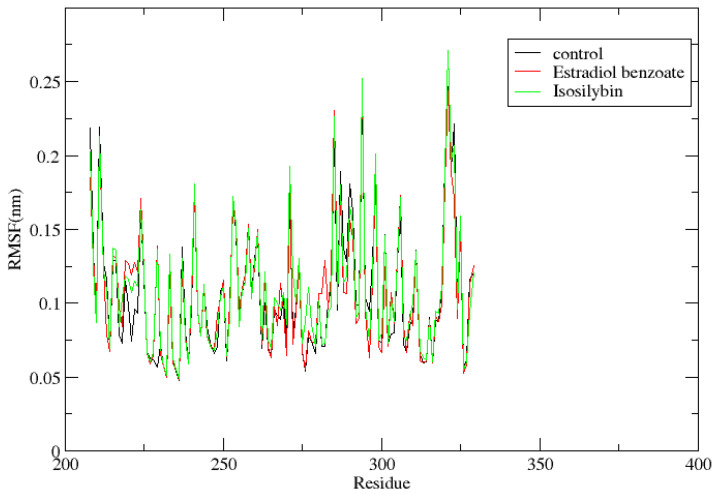
The RMSF of VP35 of Marburg virus residue bound to compounds Estradiol benzoate and Isosilybin along with control, myricetin over the 100 ns simulation.

It provides information about the fluctuations and movements of each residue relative to its average position over the course of the simulation. RMSF of the protein was computed for the 100 ns simulation for each residue that was bound to the substances isosilybin and Estradiol benzoate, as well as the control, myricetin. The RMSF for the protein is displayed in Figure 8. There were very few residues that had RMSF values greater than 2.0 nm, but none of them had RMSF values greater than 0.3 nm. Protein bound to the control, myricetin, had six residues with fluctuations of more than 0.2 nm, whereas protein bound to Estradiol benzoate and isosilybin had four and seven residues with fluctuations of more than 0.2 nm, respectively. In order to evaluate the degree of flexibility or mobility exhibited by protein residues, RMSF is applied. A lower RMSF value indicates more rigid or stable residues, whereas a higher RMSF value indicates that the residues are more mobile or flexible. Residues with higher RMSF values tend to have larger deviations from their average positions, indicating greater flexibility and conformational changes. Here, the lower RMSF indicated that the protein-ligand complexes for the compounds Estradiol benzoate and Isosilybin were stable, showing strong binding.

#### 3.4.4. Hydrogen Bond

**Figure 9:**
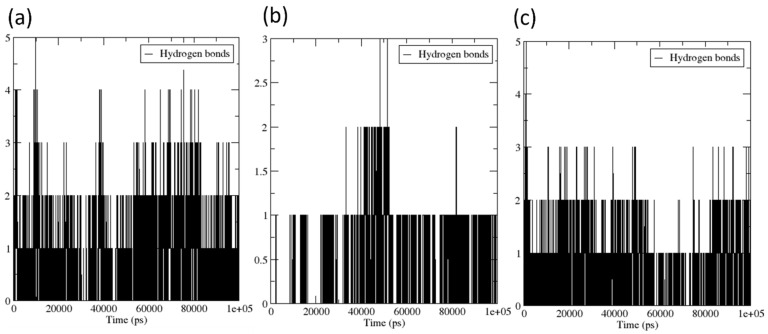
Hydrogen bonds that were generated between proteins attached to (**a**) Myricetin (the control), (**b**) estradiol benzoate, and (**c**) Isosilybin throughout the 100 ns simulation.

The formation, breakage, and fluctuations of hydrogen bonds between a protein and a ligand are examples of dynamic interactions that take place over the course of molecular dynamics (MD) simulations. MD simulations provide a thorough portrayal of the molecular movements and interactions that take place within a system. This depiction includes the hydrogen bonds that are formed between the protein and the ligand. Throughout the simulation, the role that hydrogen bonds play in preserving the stability of the protein-ligand complex is critically important. They contribute to the binding that occurs between the ligand and the protein, which prevents the two from becoming separated. Monitoring the occupancy of hydrogen bonds over time frame provides vital insights into the stability and dynamics of the complex. The hydrogen bonds that are generated between the protein VP35 of the Marburg virus and the compounds Estradiol benzoate, isosilybin, and the control (myricetin) are depicted in Figure 8. Myricetin exhibited 1 to 2 hydrogen bonds with low fluctuations (more stability), as shown in Figure 9a. On the other hand, the compounds Estradiol benzoate and Isosilybin formed single hydrogen bond each, as depicted in Figure 9b,c, respectively. During the course of the simulation, however, these compounds exhibited a significant degree of variability in the number of hydrogen bonds. Low levels of hydrogen bond count fluctuation are indicative of a more stable H-bond formation. Conversely, high levels of hydrogen bond count fluctuation during the simulation indicate dynamic structural changes. In the case of estradiol benzoate and Isosilybin, relatively low fluctuation was observed, indicating a consistent concentration of hydrogen bonds that endured throughout the 100 ns simulation.

#### 3.4.5. SASA (Solvent Accessible Surface Area)

**Figure 10:**
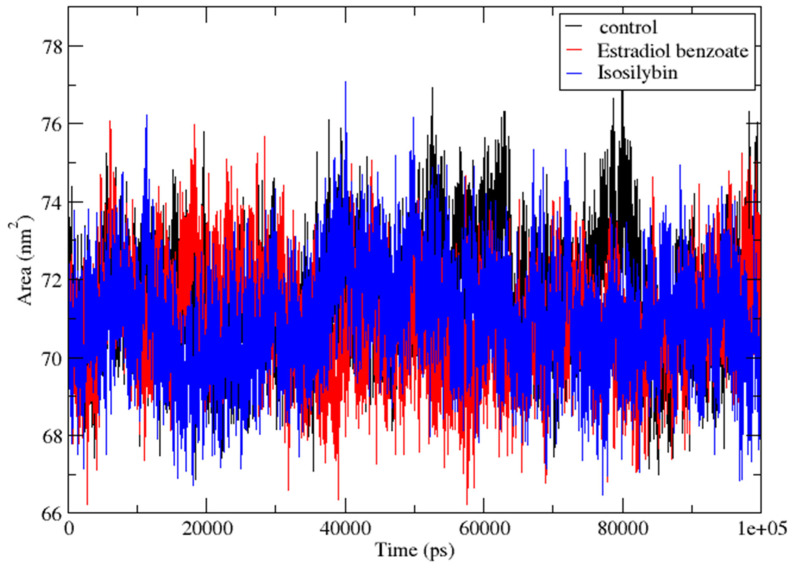
SASA (Solvent Accessible Surface Area) of the protein bound to Myricetin (control), Estradiol benzoate and Isosilybin during the 100 ns simulation.

A biomolecule’s Solvent Accessible Surface Area (SASA) is a quantitative measure of the surface area of the biomolecule that is accessible to molecules of the solvent. It reveals important insights about the surface of the molecule and how it interacts with the surrounding environment, particularly the solvent. SASA calculations, when applied to a protein-ligand complex that is going through a molecular dynamics (MD) simulation, allow for the assessment of solvent accessibility for the protein molecule. This, in turn, makes it possible to monitor protein interactions with the solvent as the simulation progresses. In this investigation, the SASA was calculated for the protein VP35 when it was complexed with the compounds Estradiol benzoate and isosilybin, in addition to the control compound myricetin. The SASA values that were determined for these complexes are presented in Figure 10. Interestingly, the SASA values were found to be highly comparable between the protein bound to the control compound and the protein-ligand complexes involving Estradiol benzoate and Isosilybin. The similarity in SASA values suggests that the binding of Estradiol benzoate and Isosilybin to the protein VP35 did not significantly alter the solvent accessibility of the protein. Based on this discovery, it may be deduced that these compounds have the potential to interact with the protein without producing significant changes in the surface exposure of the protein to the environment it is in.

Stable SASA values in protein-ligand complexes are consistent with a binding relationship that is robust and well-maintained between the protein and the ligand. In addition to this, it is an indication that the general shape of the complex is preserved all the way through the simulation or the experimental observation. Because of its stability, it appears that the protein and the ligand combine to form a strong complex. This complex has positive interactions and a low propensity to become dissociated.

### 3.5. Binding Free Energy (MM/GBSA)

In order to determine the binding free energy of the top three hits, the gmx_MMPBSA_tool was utilized [43,44]. The visualization tool known as gmx_MMPBSA_ana was utilized to plot the graphs and conduct an analysis on the energy components of the MM/GBSA. Calculations have were here to determine the binding free energy for the final 20 ns of the simulation.

**Figure 11:**
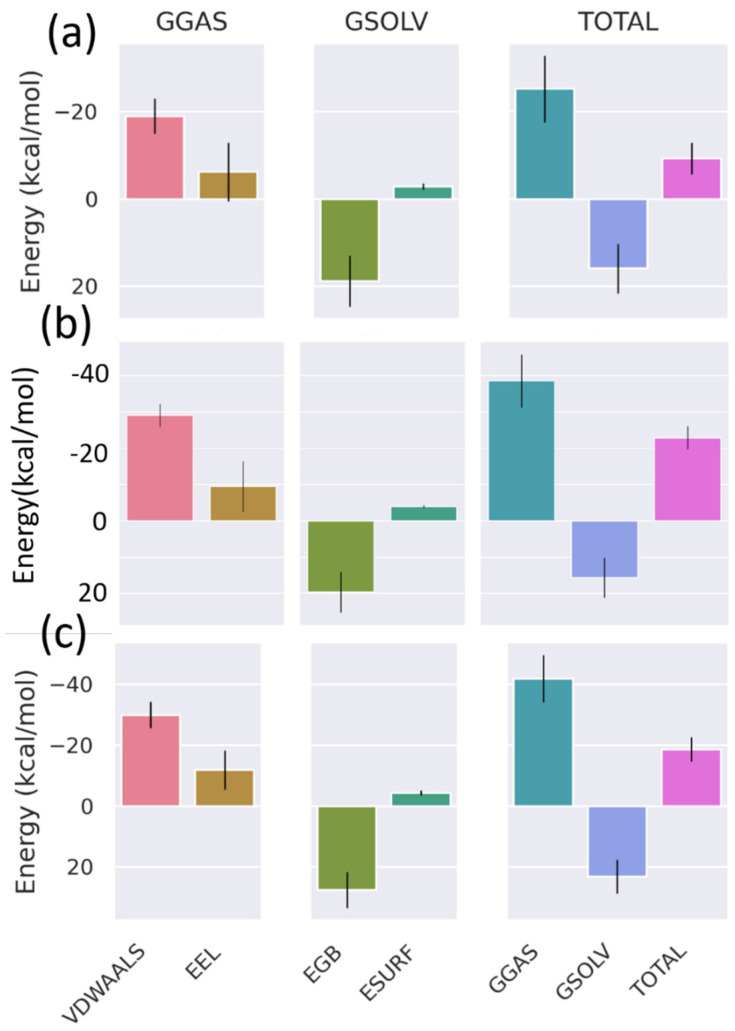
Binding free energy components computed based on MM/GBSA analysis for the ligands (**a**) Myricetin (control), (**b**) Estradiol benzoate, and (**c**) Isosilybin.

The individual energy values (E_EL_, E_VDWAALS_) and the summed (G_GAS_, G_SOLV_, and G_TOTAL_) components are depicted as bar graphs in Figure 11. Figure 11 exhibits not only the G_GAS_ but also the G_SOLV_ for each of the three complexes, which include E_EL_, E_VDWAALS_, E_GB_, and E_SURF_. According to Figure 11b, the chemical known as Estradiol benzoate displayed the lowest value for its ΔG_TOTAL_ binding free energy, which was at −22.89 kcal/mol. Figure 11 illustrates that the chemical isosilybin had a ΔG_TOTAL_ binding free energy of −18.65 kcal/mol, as shown in 11c. ΔGTOTAL binding free energy, which calculates the changes in total binding free energy due to the formation of the complex, is an important parameter for determining how ligands bind to proteins. In this case, the ΔGTOTAL binding free energy indicated that Estradiol benzoate displayed preferential binding in comparison to the compound isosilybin. In addition, the MM/GBSA analysis was used to determine the binding affinity of the reference ligand, Myricetin. The results of this analysis are presented in Figure 11a. Over the last 20 ns, the ΔG_TOTAL_ binding free energy of Myricetin revealed a value of −9.29 kcal/mol. Furthermore, when compared to Myricetin, both hit compounds (Estradiol benzoate and Isosilybin) exhibited greater ΔG_TOTAL_ binding free energy. However, when compared to Isosilybin, Estradiol benzoate showed the best ΔG_TOTAL_ binding free energy. This suggests that the two hit compounds had a higher binding affinity for the VP35 protein than the well-established reference ligand did.

**Figure 12:**
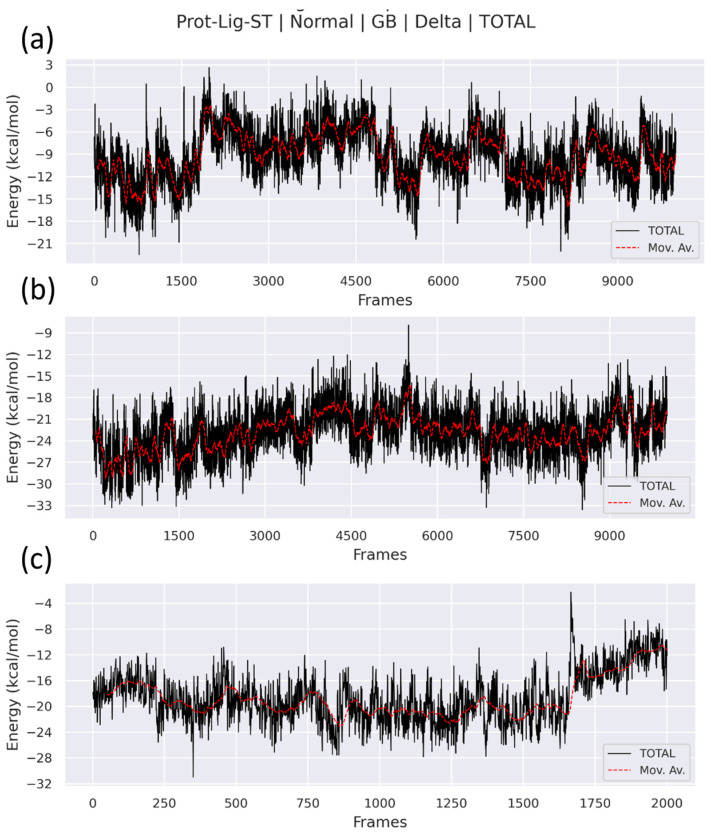
The ΔG_TOTAL_ total binding free energy plots and the running average (red line) was plotted from gmx_MMPBSA analysis for, (**a**) Myricetin (control), (**b**) Estradiol benzoate, and (**c**) Isosilcybin.

In addition, the change in total binding free energy, denoted by ΔG_TOTAL_, was displayes in Figure 12 alongside a running average, represented by the line in red. Figure 12 displayed the running average of ΔG_TOTAL_ along with the 200 frames that were collected based on the results of the MD simulation’s final 20 ns. The red line with dashes depicted the moving average of the ΔG_TOTAL_. It was observed that the Myricetin (control) had relatively high fluctuations in the running average of ΔG_TOTAL_, as shown in Figure 12a, whereas the Estradiol benzoate and isosilybin had relatively lower stable and consistent ΔG_TOTAL_ throughout the course of the final 20 ns of the simulation (Figure 12b,c, respectively). The findings of the study showed that the selected compounds, Estradiol benzoate and isosilybin, had a higher affinity toward protein when compared to the control. This was demonstrated by the fact that they had a higher correlation with protein (Myricetin). On the other hand, as compared to isosilybin, Estradiol benzoate exhibited a ΔG_TOTAL_ that was more steady, stable, and consistent. In this study, it was demonstrated that Estradiol benzoate possessed the least amount of fluctuation and, as a result, more stable binding free energy with the greatest amount of minimum ΔG_TOTAL_. Estradiol benzoate emerged as the most promising hit chemical for VP35 binding.

### 3.6. Principal Component Analysis

**Figure 13:**
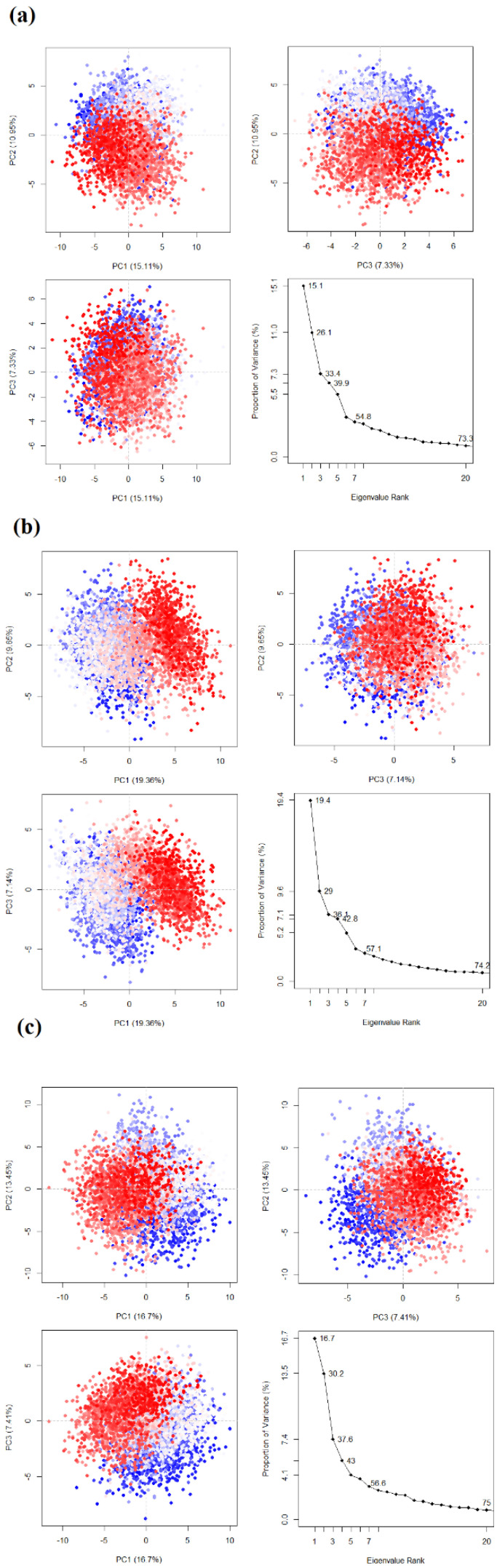
The principal component analysis (PCA) of the three complexes (**a**) Myricetin (control), (**b**) Estradiol benzoate, and (**c**) Isosilcybin.

The movement of proteins in a solvent environment is characterized by high-dimensional motion occurring in multiple directions. Principal component analysis (PCA) is a technique used to reduce the dimensionality of this motion and interpret the variations involved. In this study, the Bio3D tool was utilized to identify the first three principal components of protein motion. The analysis was performed on the entire 100 nanosecond simulation trajectory, as depicted in Figure 13. To compare the variations in protein motion upon ligand binding, a control compound called Myricetin was used. The first principal component of the control ligand, Myricetin, accounted for 15.1% of the conformational variance. In contrast, for Estradiol benzoate, it was 19.4%, and for Isosilcybin, it was 16.7%. According to these findings, the conformational variation in Estradiol benzoate was lower than that of the control compound. This indicates that the protein-ligand complex is more stable. However, when considering the cumulative effect of the first three components, Isosilcybin exhibited the highest coverage of variance, amounting to 37.6%. The variance coverage data from the first three principal components demonstrated competitive fluctuations compared to the control compound (Myricetin) system, specifically in relation to the systems complexed with Estradiol benzoate and Isosilcybin. In the PCA plots, the colors represent the time progression of the simulation, with blue indicating the initial phase and red indicating the end phase. Each dot in the plots corresponds to a single conformation. Notably, the PCA plots shown in Figure 12 exhibit similar dispersion characteristics. The overlapping of blue and red dots in the control ligand and Isosilcybin complexes suggests similarities in conformational changes between the initial and end phases of the simulation. In contrast, the colors in the Estradiol benzoate complex are relatively separated, indicating a distinction between the initial phase simulation and the end phase.

This study assessed the potential of natural small molecules to interact with the viral target protein VP35 and interfere with the viral mechanism using drug in-silico approaches. The viral protein known as VP35 is necessary for the assembly of the nucleocapsid, replication, and transcription of the virus, making it a suitable candidate for pharmaceutical development. In this study, a virtual screening approach including molecular docking, MD simulation, MM/GBSA, and PCA was employed on a natural compound library. The study suggested that Estradiol benzoate had the potential to inhibit and disrupt the functionality of VP35 belonging to the Marburg virus.

Estradiol benzoate (EB) is a pro-Estradiol hormone that binds to the estrogen receptor (ER) and its subtypes ERα and ERβ [51]. Estradiol is a naturally occurring hormone that circulates endogenously within the human body. Estradiol, the primary female sex hormone, is the most powerful estrogenic steroid found in mammals [52]. Hormone therapy, gynaecological disorder treatment [53], and the elimination of prostate cancer [54] are the most common applications of EB in female patients. Accordingly, Estradiol benzoate has been reported in numerous instances where it has been used as a competitive drug to inhibit viral growth. The antiviral activity of Estradiol benzoate against SARS-CoV-2 and the hepatitis B virus has been previously reported [52,55,56]. By binding to and disrupting the six-helix (6-HB) fusion core of SARS-CoV-2 S protein, Estradiol benzoate is able to block viral entry [56]. Using HepG2.2.15 cells and HepG2 transfected cells as a cellular model of HBV replication, it was also found that Estradiol benzoate inhibited HBV replication and transcription [55].

The current study showed the significance of Estradiol benzoate as a potential therapeutic option against the Marburg virus. Intermolecular interactions between Estradiol benzoate and VP35 revealed a carbon-hydrogen bond (covalent interaction) with the residue Pro^293^, showing a significant interaction with the protein. Furthermore, the results of molecular docking in this study showed that Estradiol benzoate had a binding energy of −8.1 kcal/mol with VP35. The consistent RMSD during the last 60 ns MD simulations confirmed that Estradiol benzoate had a stable confirmation with the protein binding site. MD simulation results indicated that Estradiol benzoate with VP35 had the most favorable ΔG (−22.89 kcal/mol).

Additionally, an analysis of the absorption, distribution, metabolism, excretion, and toxicity (ADMET) properties was performed to study the drug suitability, toxicity, and drug-likeness of Estradiol benzoate, as shown in supplementary Table S2. This study suggested that Estradiol benzoate had all the favorable characteristics of a drug according to the Lipinski rule. It had acceptable molecular weight (MW), H-bond acceptors and H-bond donors, and iLogP values. It could pass in vitro and in vivo studies without triggering a PAINS alert. It belonged to the acceptable toxicity class of 5. These findings indicate drug-like properties of Estradiol benzoate, in particular regarding their potential for oral bioavailability. Overall, this investigation suggested that Estradiol benzoate possesses favorable attributes, indicating a potential inhibitory mechanism against the VP35 protein of the Marburg virus.

## 4. Conclusions

As there is currently no specific antiviral treatment available for Marburg virus infection, the primary form of treatment remains supportive care. Outbreaks of Marburg virus disease are rare but can have a high mortality rate, ranging from 23% to 90%, depending on the outbreak and healthcare resources available. The search for an antiviral compound against Marburg virus is of paramount importance due to the severe and often fatal nature of Marburg virus disease. VP35 RNA binding can be used as a potential drug target for MARV as it is effective in Ebola infection treatment. Among the selected screened compounds in this study, Estradiol benzoate, INVEGA (Paliperidone), and Isosilybin showed hydrogen bond formation with the protein, while the other compounds showed none.

Molecular dynamics (MD) simulation was performed on Estradiol benzoate, INVEGA (Paliperidone), and Isosilybin, along with the control Myricetin. However, INVEGA (Paliperidone) could not remain in the binding pocket. The RMSD of the ligands revealed that Myricetin, which served as a control, had the most consistent RMSD, while Estradiol benzoate demonstrated consistent RMSD, and Isosilybin demonstrated significant RMSD fluctuation. In comparison, Estradiol benzoate demonstrated a binding with the protein that was remarkably stable for the majority of the time that the simulation was run. Based on the root mean square deviation (RMSD), the MD simulation analysis determined that the ligand Estradiol benzoate had higher stability in comparison. The MM/GBSA analysis on the trajectory reconfirmed the results through ΔGTOTAL binding free energy that ligand Estradiol benzoate had the most stable complex, and thus this compound should be taken further for in vitro studies to validate its inhibitory action. Estradiol benzoate had a strong binding energy of −8.1 kcal/mol with VP35, while it also had a significant hydrogen bond (covalent interaction) with the residue Pro293. Both stable RMSD and the lowest binding free energy of Estradiol benzoate advocate it as a therapeutic candidate for Marburg virus. The ADMET properties of Estradiol benzoate also suggest it as a promising candidate for further exploration. Overall, this study proposed Estradiol benzoate as a promising binder for the VP35 RNA-binding domain of Marburg virus and could be further investigated in future experiments.

## Data Availability

The datasets used and/or analyzed during the current study are available from the corresponding author on reasonable request.

## Supplementary Materials

The following supporting information can be downloaded at: https://www.mdpi.com/article/10.3390/v15081739/s1, Table S1: Parameters and settings used for MD simulation during NVT, NPT and MD production run.; Table S2: Table S1. The ADME-toxicity study of Estradiol benzoate using SwissADME and ProTox-II (Prediction of Toxicity of Chemicals) servers.

## References

[1] Marburg Virus Disease. https://www.who.int/news-room/fact-sheets/detail/marburg-virus-disease.

[2] Kuhn J.H., Amarasinghe G.K., Basler C.F., Bavari S., Bukreyev A., Chandran K., Crozier I., Dolnik O., Dye J.M., Formenty P.B.H. (2019). ICTV Virus Taxonomy Profile: Filoviridae. J. Gen. Virol..

[3] Towner J.S., Pourrut X., Albariño C.G., Nkogue C.N., Bird B.H., Grard G., Ksiazek T.G., Gonzalez J.-P., Nichol S.T., Leroy E.M. (2007). Marburg Virus Infection Detected in a Common African Bat. PLoS ONE.

[4] Kwiecinski G.G., Kwiecinski G.G., Griffiths T.A. (1999). Rousettus Egyptiacus. Mamm. Species.

[5] Almeida F.C., Giannini N.P., Simmons N.B. (2016). The Evolutionary History of the African Fruit Bats (Chiroptera: Pteropodidae). Acta Chiropterologica.

[6] Gordon Smith C.E., Simpson D.I.H., Bowen E.T.W., Zlotnik I. (1967). Fatal Human Disease from Vervet Monkeys. Lancet.

[7] Kissling R.E., Robinson R.Q., Murphy F.A., Whitfield S.G. (1968). Agent of Disease Contracted from Green Monkeys. Science.

[8] Swanepoel R., Smit S.B., Rollin P.E., Formenty P., Leman P.A., Kemp A., Burt F.J., Grobbelaar A.A., Croft J., Bausch D.G. (2007). Studies of Reservoir Hosts for Marburg Virus. Emerg. Infect. Dis..

[9] Towner J.S., Khristova M.L., Sealy T.K., Vincent M.J., Erickson B.R., Bawiec D.A., Hartman A.L., Comer J.A., Zaki S.R., Ströher U. (2006). Marburgvirus Genomics and Association with a Large Hemorrhagic Fever Outbreak in Angola. J. Virol..

[10] Marburg Virus Disease—Equatorial Guinea. https://www.who.int/emergencies/disease-outbreak-news/item/2023-DON449.

[11] Marburg Virus Disease—United Republic of Tanzania. https://www.who.int/emergencies/disease-outbreak-news/item/2023-DON451.

[12] Marzi A., Feldmann H. (2023). Marburg Virus Disease: Global Threat or Isolated Events?. J. Infect. Dis..

[13] Hickman M.R., Saunders D.L., Bigger C.A., Kane C.D., Iversen P.L. (2022). The Development of Broad-Spectrum Antiviral Medical Countermeasures to Treat Viral Hemorrhagic Fevers Caused by Natural or Weaponized Virus Infections. PLoS Negl. Trop. Dis..

[14] Bradfute S.B. (2022). The Discovery and Development of Novel Treatment Strategies for Filoviruses. Expert Opin. Drug Discov..

[15] Bukreyev A.A., Volchkov V.E., Blinov V.M., Dryga S.A., Netesov S.V. (1995). The Complete Nucleotide Sequence of the Popp (1967) Strain of Marburg Virus: A Comparison with the Musoke (1980) Strain. Arch. Virol..

[16] Leung D.W., Prins K.C., Basler C.F., Amarasinghe G.K. (2010). Ebolavirus VP35 Is a Multifunctional Virulence Factor. Virulence.

[17] Johnson R.F., McCarthy S.E., Godlewski P.J., Harty R.N. (2006). Ebola Virus VP35-VP40 Interaction Is Sufficient for Packaging 3E-5E Minigenome RNA into Virus-Like Particles. J. Virol..

[18] Haasnoot J., Vries W., de Geutjes E.-J., Prins M., de Haan P., Berkhout B. (2007). The Ebola Virus VP35 Protein Is a Suppressor of RNA Silencing. PLoS Pathog..

[19] Basler C.F., Mikulasova A., Martinez-Sobrido L., Paragas J., Mühlberger E., Bray M., Klenk H.-D., Palese P., García-Sastre A. (2003). The Ebola Virus VP35 Protein Inhibits Activation of Interferon Regulatory Factor 3. J. Virol..

[20] Hartman A.L., Dover J.E., Towner J.S., Nichol S.T. (2006). Reverse Genetic Generation of Recombinant Zaire Ebola Viruses Containing Disrupted IRF-3 Inhibitory Domains Results in Attenuated Virus Growth In Vitro and Higher Levels of IRF-3 Activation without Inhibiting Viral Transcription or Replication. J. Virol..

[21] Schümann M., Gantke T., Mühlberger E. (2009). Ebola Virus VP35 Antagonizes PKR Activity through Its C-Terminal Interferon Inhibitory Domain. J. Virol..

[22] Liu G., Nash P.J., Johnson B., Pietzsch C., Ilagan M.X.G., Bukreyev A., Basler C.F., Bowlin T.L., Moir D.T., Leung D.W. (2017). A Sensitive in Vitro High-Throughput Screen To Identify Pan-Filoviral Replication Inhibitors Targeting the VP35–NP Interface. ACS Infect. Dis..

[23] Hasan M., Mia M.M., Islam M.M., Hasan Saraf M.S., Islam M.S. (2022). A Computerized Pharmaceutical Repurposing Approach Reveals Semicochliodinol B Synthesized from Chrysosporium Merdarium as a Viable Therapeutic Contender for Marburg Virus’s VP35 and VP40 Proteins. Inform. Med. Unlocked.

[24] Bale S., Julien J.-P., Bornholdt Z.A., Kimberlin C.R., Halfmann P., Zandonatti M.A., Kunert J., Kroon G.J.A., Kawaoka Y., MacRae I.J. (2012). Marburg Virus VP35 Can Both Fully Coat the Backbone and Cap the Ends of DsRNA for Interferon Antagonism. PLoS Pathog..

[25] Heikamp K., Bajorath J. (2013). The Future of Virtual Compound Screening. Chem. Biol. Drug Des..

[26] Lill M., Kortagere S. (2013). Virtual Screening in Drug Design. In Silico Models for Drug Discovery.

[27] Fischer A., Smieško M., Sellner M., Lill M.A. (2021). Decision Making in Structure-Based Drug Discovery: Visual Inspection of Docking Results. J. Med. Chem..

[28] Eberhardt J., Santos-Martins D., Tillack A.F., Forli S. (2021). AutoDock Vina 1.2.0: New Docking Methods, Expanded Force Field, and Python Bindings. J. Chem. Inf. Model..

[29] El-Demerdash A., Al-Karmalawy A.A., Abdel-Aziz T.M., Elhady S.S., Darwish K.M., Hassan A.H.E. (2021). Investigating the Structure–Activity Relationship of Marine Natural Polyketides as Promising SARS-CoV-2 Main Protease Inhibitors. RSC Adv..

[30] Tian W., Chen C., Lei X., Zhao J., Liang J. (2018). CASTp 3.0: Computed Atlas of Surface Topography of Proteins. Nucleic Acids Res..

[31] Nath A., Kumer A., Khan M.W. (2021). Synthesis, Computational and Molecular Docking Study of Some 2, 3-Dihydrobenzofuran and Its Derivatives. J. Mol. Struct..

[32] Cosconati S., Forli S., Perryman A.L., Harris R., Goodsell D.S., Olson A.J. (2010). Virtual Screening with AutoDock: Theory and Practice. Expert Opin. Drug Discov..

[33] Nath A., Kumer A., Zaben F., Khan M.W. (2021). Investigating the Binding Affinity, Molecular Dynamics, and ADMET Properties of 2,3-Dihydrobenzofuran Derivatives as an Inhibitor of Fungi, Bacteria, and Virus Protein. Beni-Suef Univ. J. Basic Appl. Sci..

[34] Rahman M.M., Islam M.R., Akash S., Mim S.A., Rahaman M.S., Emran T.B., Akkol E.K., Sharma R., Alhumaydhi F.A., Sweilam S.H. (2022). In Silico Investigation and Potential Therapeutic Approaches of Natural Products for COVID-19: Computer-Aided Drug Design Perspective. Front. Cell. Infect. Microbiol..

[35] Biovia D.S. (2015). Discovery Studio Modeling Environment.

[36] Cichero E., Calautti A., Francesconi V., Tonelli M., Schenone S., Fossa P. (2021). Probing In Silico the Benzimidazole Privileged Scaffold for the Development of Drug-like Anti-RSV Agents. Pharmaceuticals.

[37] Gelpi J., Hospital A., Goñi R., Orozco M. (2015). Molecular Dynamics Simulations: Advances and Applications. Adv. Appl. Bioinforma. Chem..

[38] Vanommeslaeghe K., Raman E.P., MacKerell A.D. (2012). Automation of the CHARMM General Force Field (CGenFF) II: Assignment of Bonded Parameters and Partial Atomic Charges. J. Chem. Inf. Model..

[39] Darden T., York D., Pedersen L. (1993). Particle Mesh Ewald: An N⋅log(N) Method for Ewald Sums in Large Systems. J. Chem. Phys..

[40] Hess B., Bekker H., Berendsen H.J.C., Fraaije J.G.E.M. (1997). LINCS: A Linear Constraint Solver for Molecular Simulations. J. Comput. Chem..

[41] Bussi G., Donadio D., Parrinello M. (2007). Canonical Sampling through Velocity Rescaling. J. Chem. Phys..

[42] Parrinello M., Rahman A. (1981). Polymorphic Transitions in Single Crystals: A New Molecular Dynamics Method. J. Appl. Phys..

[43] Valdés-Tresanco M.S., Valdés-Tresanco M.E., Valiente P.A., Moreno E. (2021). Gmx_MMPBSA: A New Tool to Perform End-State Free Energy Calculations with GROMACS. J. Chem. Theory Comput..

[44] Miller B.R.I., McGee T.D., Swails J.M., Homeyer N., Gohlke H., Roitberg A.E. (2012). MMPBSA.Py: An Efficient Program for End-State Free Energy Calculations. J. Chem. Theory Comput..

[45] Skjærven L., Yao X.-Q., Scarabelli G., Grant B.J. (2014). Integrating Protein Structural Dynamics and Evolutionary Analysis with Bio3D. BMC Bioinformatics.

[46] Grant B.J., Rodrigues A.P.C., ElSawy K.M., McCammon J.A., Caves L.S.D. (2006). Bio3d: An R Package for the Comparative Analysis of Protein Structures. Bioinformatics.

[47] Jolliffe I.T. (2002). Principal Component Analysis for Special Types of Data. Principal Component Analysis.

[48] Daina A., Michielin O., Zoete V. (2017). SwissADME: A Free Web Tool to Evaluate Pharmacokinetics, Drug-Likeness and Medicinal Chemistry Friendliness of Small Molecules. Sci. Rep..

[49] Banerjee P., Eckert A.O., Schrey A.K., Preissner R. (2018). ProTox-II: A Webserver for the Prediction of Toxicity of Chemicals. Nucleic Acids Res..

[50] Karplus M., McCammon J.A. (2002). Molecular Dynamics Simulations of Biomolecules. Nat. Struct. Biol..

[51] Refaie M.M.M., El-Hussieny M. (2017). Diacerein Inhibits Estradiol-Benzoate Induced Cervical Hyperkeratosis in Female Rats. Biomed. Pharmacother. Biomedecine Pharmacother..

[52] Mahdian S., Zarrabi M., Panahi Y., Dabbagh S. (2021). Repurposing FDA-Approved Drugs to Fight COVID-19 Using in Silico Methods: Targeting SARS-CoV-2 RdRp Enzyme and Host Cell Receptors (ACE2, CD147) through Virtual Screening and Molecular Dynamic Simulations. Inform. Med. Unlocked.

[53] Israel G.E., Tarver D.E. (1997). Transgender Care: Recommended Guidelines, Practical Information, and Personal Accounts.

[54] Recent Advances on Bisphenol-A and Endocrine Disruptor Effects on Human Prostate Cancer—PubMed. https://pubmed.ncbi.nlm.nih.gov/28257827/.

[55] He J., Wu J., Chen J., Zhang S., Guo Y., Zhang X., Han J., Zhang Y., Guo Y., Lin Y. (2022). Identification of Estradiol Benzoate as an Inhibitor of HBx Using Inducible Stably Transfected HepG2 Cells Expressing HiBiT Tagged HBx. Molecules.

[56] Yang C., Pan X., Huang Y., Cheng C., Xu X., Wu Y., Xu Y., Shang W., Niu X., Wan Y. (2021). Drug Repurposing of Itraconazole and Estradiol Benzoate against COVID-19 by Blocking SARS-CoV-2 Spike Protein-Mediated Membrane Fusion. Adv. Ther..

