# Supplementary material for: Cheminformatics Strategies Unlock Marburg Virus VP35 Inhibitors from Natural Compound Library
Source: Viruses. 2023 Aug 15;15(8):1739. doi: 10.3390/v15081739 (PMC10459822; doi:10.3390/v15081739)
Supplement: Supplementary file 1 [file viruses-15-01739-s001.zip › viruses-2532024-supplementary.pdf]

Supplementary

Table S1. Parameters and settings used for MD simulation during NVT, NPT and MD production run.

**NVT**	**Cut-off**	**NPT**	**Cut-off**	**MD run**	**Cut-off**
**nsteps**	50000	nsteps	500000	nsteps	50000000
dt	0.002	dt	0.002	dt	0.002
nstenergy	500	nstenergy	500	nstxout	0
nstlog	500	nstlog	500	nstvout	0
nstxout-compressed	500	nstxout-compressed	500	nstxtcout	1000
lincs_iter	1	lincs_iter	1	nstenergy	1000
lincs_order	4	lincs_order	4	nstlog	1000
nstlist	20	nstlist	20	lincs_iter	1
rlist	1.2	rlist	1.2	lincs_order	4
rvdw-switch	1	rvdw-switch	1	nstlist	5
rvdw	1.2	rvdw	1.2	rlist	1.2
rcoulomb	1.2	rcoulomb	1.2	rlistlong	1.2
pme_order	4	pme_order	4	rvdw-switch	1
fourierspacing	0.16	fourierspacing	0.16	rcoulomb	1.2
tau_t	0.1	tau_t	0.1	rvdw	1.2
ref_t	310	ref_t	310	pme_order	4
gen_temp	310	tau_p	2	fourierspacing	0.16
gen_seed	-1	ref_p	1	tau_t	0.1
		compressibility	4.50e-05	ref_t	310
				tau_p	4
				ref_p	1
				compressibility	4.50e-05

Table S2. The ADME-toxicity study of Estradiol benzoate using SwissADME and ProTox-II (Prediction of Toxicity of Chemicals) servers.

ADMET	Estradiol benzoate
**MW**	376.49
Rotatable bonds	3
H-bond acceptors	3
H-bond donors	1
MR	110.4
TPSA	46.53
iLOGP	3.9
ESOL Class	Moderately soluble
**PAINS alerts**	0
Predicted Toxicity Class	5
